# Removal of Nitrogen and Phosphorus from Thickening Effluent of an Urban Wastewater Treatment Plant by an Isolated Green Microalga

**DOI:** 10.3390/plants9121802

**Published:** 2020-12-18

**Authors:** Costanza Baldisserotto, Sara Demaria, Ornella Accoto, Roberta Marchesini, Marcello Zanella, Linda Benetti, Francesco Avolio, Michele Maglie, Lorenzo Ferroni, Simonetta Pancaldi

**Affiliations:** 1 Department of Life Sciences and Biotechnology, University of Ferrara, C.so Ercole I d’Este, 32, 44121 Ferrara, Italy; costanza.baldisserotto@unife.it (C.B.); sara.demaria@unife.it (S.D.); ornella.accoto@gmail.com (O.A.); roberta.marchesini@unife.it (R.M.); michele.maglie@unife.it (M.M.); lorenzo.ferroni@unife.it (L.F.); 2 HERA SpA—Direzione Acqua, Via C. Diana, 40, Cassana, 44044 Ferrara, Italy; Marcello.Zanella@gruppohera.it (M.Z.); Linda.Benetti@gruppohera.it (L.B.); Francesco.Avolio@gruppohera.it (F.A.)

**Keywords:** nutrient removal, nitrogen, phosphorus, urban wastewaters, autochthonous microalgae

## Abstract

Microalgae are photosynthetic microorganisms and are considered excellent candidates for a wide range of biotechnological applications, including the removal of nutrients from urban wastewaters, which they can recover and convert into biomass. Microalgae-based systems can be integrated into conventional urban wastewater treatment plants (WW-TP) to improve the water depuration process. However, microalgal strain selection represents a crucial step for effective phytoremediation. In this work, a microalga isolated from the effluent derived from the thickening stage of waste sludge of an urban WW-TP was selected and tested to highlight its potential for nutrient removal. Ammonium and phosphate abatements by microalgae were evaluated using both the effluent and a synthetic medium in a comparative approach. Parallelly, the isolate was characterized in terms of growth capability, morphology, photosynthetic pigment content and photosystem II maximum quantum yield. The isolated microalga showed surprisingly high biomass yield and removal efficiency of both ammonium and phosphate ions from the effluent but not from the synthetic medium. This suggests its clear preference to grow in the effluent, linked to the overall characteristics of this matrix. Moreover, biomass from microalgae cultivated in wastewater was enriched in photosynthetic pigments, polyphosphates, proteins and starch, but not lipids, suggesting its possible use as a biofertilizer.

## 1. Introduction

The rise in the world population and the increasing use of natural resources which have occurred during the last century have caused strong alterations to the environment. As it is known, the world overpopulation is gradually increasing the demand for freshwater, especially in cities, also leading to a consequent increase in wastewater production [1,2]. Urban wastewater treatment plants (WW-TP) play a central role in domestic and industrial wastewater management for safe water reuse or disposal [3,4]. Commonly, conventional municipal plants treat wastewaters biologically through the activated sludge process (ASP), which in turn produces a waste sludge (WAS, waste activated sludge) needing further treatments (thickening, digestion and dewatering) [5]. During the entire process, water, derived from WAS treatment, is usually still enriched in nutrients, especially nitrogen (N) and phosphorus (P); thus, it cannot be directly released into natural environments or reused. As a consequence, it is normally recirculated in the WW-TP for N and P abatement before discharge. In fact, N and P, once accumulated in natural water bodies (lakes, rivers, lagoons, seas), cause eutrophication, with undesirable disturbance of the balance among living organisms and, then, a reduction in water quality [6,7]. However, the availability of large amounts of nutrient-rich waters represents an opportunity to couple nutrient removal and biomass production; indeed, in the last few decades, wastewaters, because of their composition not only in macro- but also in micro-nutrients, have been widely proposed as alternative low-cost culture media for photosynthetic microorganisms, especially eukaryotic microalgae, including *Chlorella*, *Scenedesmus*, *Chlorococcum*, *Botryococcus*, *Nannochloropsis* and *Neochloris* [2,7,8,9,10,11,12,13,14,15]. Furthermore, the use of algae-based wastewater treatments represents a promising route for an environment-friendly technology for nutrient recovery [12,13,16]. Microalgae are, in fact, photosynthetically active microorganisms which use solar energy and CO_2_ to reduce inorganic nutrients to organic matter, i.e., biomass, and are O_2_-evolving organisms, thus mitigating the negative impact of CO_2_ emissions, especially in high CO_2_-emitting systems such as industries and WW-TPs [7,17]. On the whole, microalgae-based processes for the phytoremediation of wastewaters have gained growing attention owing to at least five advantages: 1. simultaneous N and P removal from water with consequent conversion into microalgal biomass; 2. several potential uses of the harvested biomass; 3. high growth rates; 4. cost-effective and environment-friendly cultivation, since no addition of chemicals and no or low addition of freshwater are required, while oxygen generation, CO_2_ mitigation and metal reduction can take place at the same time; 5. the effluent discharged in the water bodies is oxygenated [6,7].

Microalgae are regarded as very good candidates for their application in WW-TPs as tertiary phytoremediation treatments of urban wastewater (UWW) effluents and for natural resource preservation, thanks to recycling processes in a “waste-to-value” approach. As aforementioned, microalgal biomass is proposed for several uses (for example, as raw material for biofertilizer, feed, food/feed supplements, biofuel) [1,2,9,18,19,20,21,22,23,24,25]. The proposed application of microalgae derived from bioremediation processes is mainly for the production of biofuels or biofertilizer, but also for feeding anaerobic biodigestion plants [1,2,9,19,22,26]. It is known that conventional WW-TPs are energy-consuming, with a resulting elevated energy bill; for example, aeration in the biological reactor alone requires 47–70% of total energy and remains a major issue in the economic management of these plants [5,27,28,29,30]. Thus, anaerobic digestion of sludge is common in WW-TPs as a method for energy recovery, and the co-digestion of sludge and microalgal biomass, harvested from integrated phytoremediation ponds, is proposed to relieve the energetic costs [5]. Whatever the final application of algal biomass, its harvesting is also a relevant issue. Flocculation and/or floatation steps are recommended to obtain clean wastewater and microalgae biomass in the form of sludge. In WW-TPs, the employment of conventional flocculants (such as chitosan, polyelectrolites, etc.) could be an effective solution, since these compounds are commonly used to improve the dewatering of WAS [31].

The choice of the most suitable microalgal organism is a crucial step in developing an effective microalgae-based tertiary treatment inside WW-TPs. Many studies have focused on the selection of the best candidates among algal strains from culture collections for this application [2,9,13,15], but concomitantly, increasing attention is being paid to the isolation of autochthonous microalgae, both as monocultures or as alga/alga or alga/bacteria consortia [26,32,33,34,35,36,37,38,39]. It is plausible that native microalgae, isolated and thus already adapted to the physico-chemical characteristics of wastewater, can be both more productive and resilient to other organisms present in the water (examples: nitrifying bacteria, pathogens, grazers, parasites) than collection strains [7,38,40]. Overall, algae well-adapted to a cultivation medium do not show signs of stress and this can also be seen from their morphology [11,21,41].

Among UWW effluents from WAS treatments, much attention has been directed towards the employment of centrate streams after the dewatering process or of the surnatant from the anaerobic digestion [22,33,38,40,42,43]. Relatively little has been, instead, reported on the employment of the streams derived from the thickening step [37,44]. Thus, it is important to further investigate the use of this wastewater for the cultivation of microalgae, both in terms of concomitant removal of N and P and of possible use as an effective culture medium for the production of algal biomass from a “waste-to-value” perspective. For example, P represents an environmental pollutant owing to its impact on eutrophication, but, at the same time, it is a component of fertilizers for the cultivation of both microalgae and crops [45]. Thus, the opportunity to valorize, through phytoremediation, waste P into algal biomass to be used as biofertilizer represents added value.

Therefore, the aim of this work was to isolate promising autochthonous microalgae from the effluent derived from the thickening step of the WW-TP located in Ferrara (Italy) to be employed in an eco-friendly phytoremediation treatment to improve the performance of the WW-TP. Ferrara is the largest city immediately next to the delta of the Po River and its regional park, which include inland and coastal wetlands of high conservation value; thus, more efficient functionality of the wastewater depuration process as a whole can represent a benefit also for the protection of the environment of such an important area. On the other hand, the use of microalgae isolated from the thickening stage of WAS treatment can also be extended to other contexts with similar problems and wastewaters. The study was planned to test algal growth and nutrient removal from the UWW effluent using the most promising microalga among those isolated or collected from the stream. For the research, microalgae were studied through morphological observations (light and transmission electron microscopy, TEM), growth analyses (cell density, biomass yield and productivity, growth rate), maximum efficiency of the photosystem II (PSII) quantum yield (F_V_/F_M_ ratio) and photosynthetic pigment content; total protein content was also determined in the final harvested biomass after the phytoremediation test with the UWW effluent. In parallel, N and P removal from the synthetic media or UWW was monitored.

The research, performed under laboratory conditions, represents the first necessary step in developing a customized prototype phytoremediation plant to be located next to the WW-TP of HERA-Ferrara. In order to adapt the research to the specific case study, in which currently there is no availability of additional purified CO_2_ supply, the experiments on phytoremediation were performed without the use of CO_2_.

## 2. Results

### 2.1. Microalgae Isolates from the UWW Effluent

**Figure 1:**
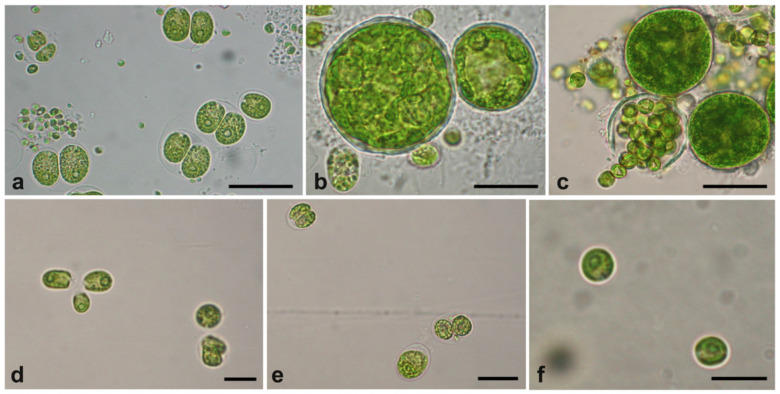
Overall view of the most characteristic microalgal forms collected from the UWW. (**a**–**d**) Microalgal forms from the upper layer; (**e**,**f**) microalgal forms from the lower layer. Bars: (**a**–**c)**, 20 µm; (**d**), (**e**), 10 µm; (**f**), 5 µm.

The light incubation process of UWW has led to the collection of a certain variety of microalgae with even very different sizes (from 3–4 up to 40–50 µm) and shapes (spherical, ovoidal, with/without flagella), the most characteristic being reported in Figure 1. In detail, samples collected from the upper layer of the incubation jars were composed of consortia containing large algal cells, mixed with smaller cells and, sometimes, bacteria (Figure 1a–c), or of flagellate forms (Figure 1d). The very large spherical cells (>40 µm, diameter) in consortia (Figure 1b,c) released, in some cases, several smaller cells, indicating a type of reproduction by autospores or zoospores (Figure 1c). Differently, the most abundant lower layer of material grown in the UWW allowed us to obtain a mixture of not-flagellate and relatively small-size algal cells: some of the microalgal material was formed by almost spherical cells (10–15 µm in size) with an external evident envelope (Figure 1e), while small, spherical cells (3–4 µm) were highly predominant in most cases (Figure 1f). 

**Figure 2:**
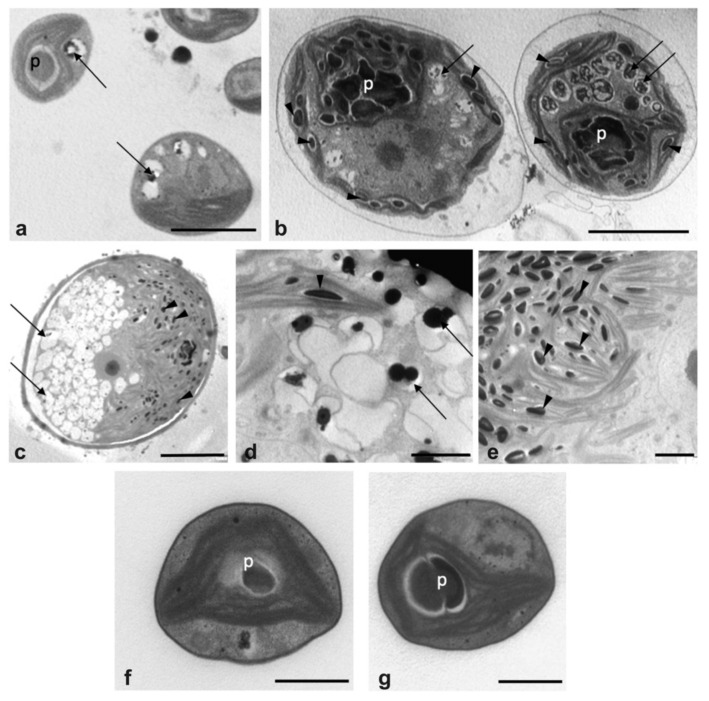
Overall view of microalgae grown in the UWW. (**a**–**e**) Algae belonging to the consortia in the upper layer of the incubation jars and (**f**,**g**) algae isolated as monoculture from the lower layer of the incubation jar. (**a**) Small cells with large chloroplast containing a pyrenoid and some small vacuolations. (**b**) Cells surrounded by an envelope and with numerous cytoplasmic vacuolations: a large chloroplast, containing a pyrenoid and numerous stromatic starch granules, is visible inside the cells. (**c**) Micrograph of a large cell with a characteristic bipartition: on the left, numerous vacuolations, and, on the right, a large chloroplast with numerous stromatic starch granules. (**d**,**e**) Magnification of the large cell in (**c**): details of vacuolations with electron-dense depositions due to polyphosphates (**d**) and of the chloroplast with bundles of thylakoids and numerous stromatic starch granules (**e**). (**f**,**g**) Cells containing an evident pyrenoid inside the very large chloroplast. (**g**) Cell showing the typical thylakoid penetrating into the pyrenoid matrix. p, pyrenoid; arrows, vacuolations; arrow heads, stromatic starch granules. Bars: (**a**), 2 µm; (**b**), 5 µm; (**c**), 10 µm; (**d**), (**e**), 2 µm; (**f**), (**g**), 1 µm.

Among the microalgae described above, consortia from the upper layer and the small spherical microalgae in the lower one were the most dominant and representative algae grown in the sewage. Thus, they were selected to be studied also at the ultrastructural level to better understand their morphology and potential for nutrient removal. TEM observations highlighted that microalgae in the consortia were featured as: 1. small cells of 2–3 µm, containing a large chloroplast with evident pyrenoid (Figure 2a); 2. larger single cells (10–15 µm), surrounded by an envelope, containing a cup-shaped chloroplast with an irregular pyrenoid and enriched in stromatic starch granules (Figure 2b); 3. very large cells (30–40 µm) (Figure 2c), with a thick cell wall and characterized by an evident bipartition at the cytoplasmic level. In the latter cells, one side of the cell was occupied by extremely abundant vacuolations (Figure 2d), the other side by a large chloroplast with thylakoids organized in bundles and rich in stromatic starch (Figure 2e). In all cell types, vacuolations contained dark precipitates, ascribable to polyphosphate depositions, suggesting an intracellular P accumulation.

Differently, the dominant algal form harvested from the lower layer was formed, as expected, by homogeneous populations constituted of only small cells (around 2–3 µm) (Figure 2f,g), similar to that in the consortia (Figure 2a) and referred to as belonging to a *Chlorella*-like genus, due to morphological characteristics, such as the chloroplast feature with a large pyrenoid [46,47,48]. In Figure 2g, typical thylakoid lamellae that penetrate into the pyrenoid matrix are recognizable.

Among the microalgal material grown in the effluent, subsequent cultivation in Petri dishes allowed the isolation of the *Chlorella*-like cells as a monoculture free of bacterial contaminants. This monoculture was selected for further studies.

### 2.2. Phytoremediation Experiment

#### 2.2.1. Comparative Growth in UWW and Modified BG11

**Figure 3:**
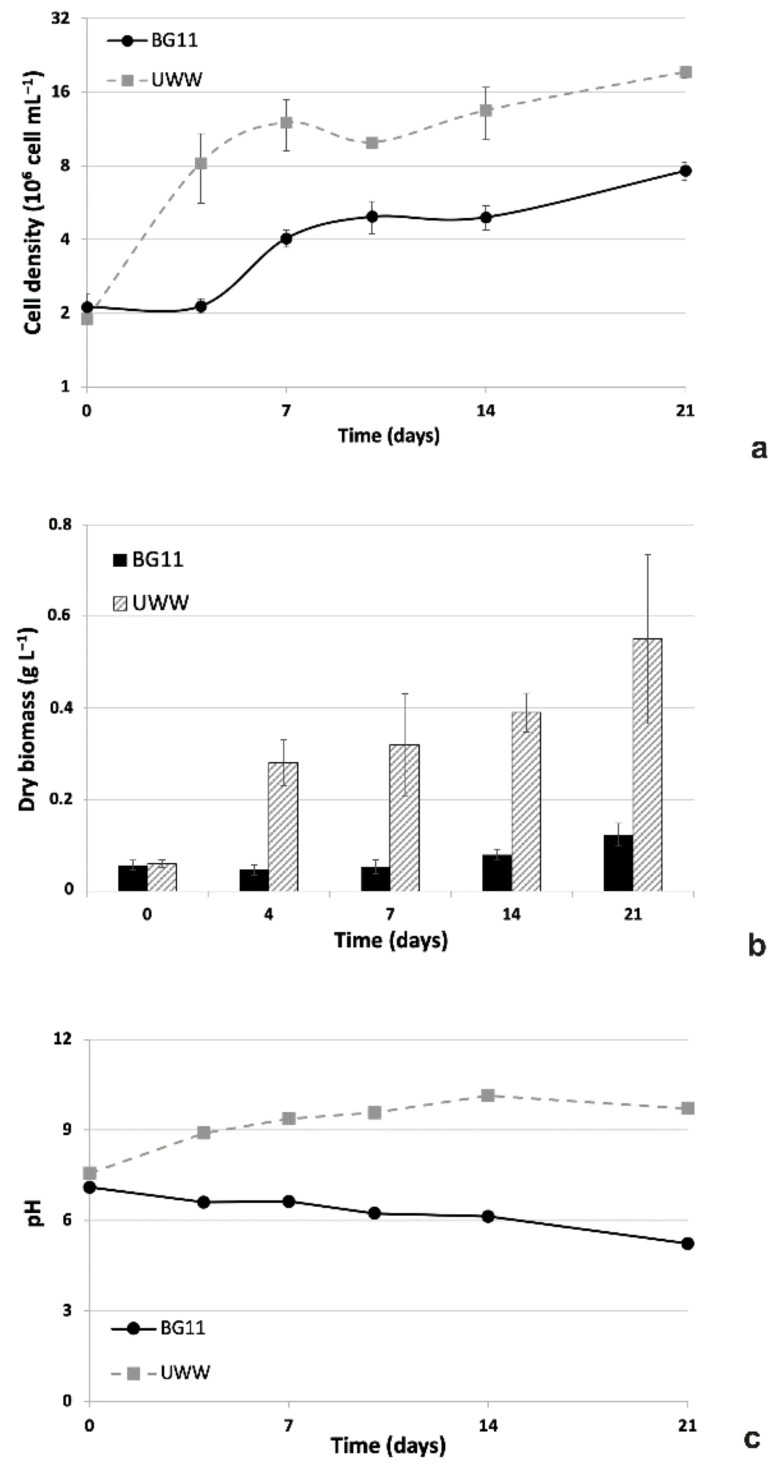
Growth parameters of microalgal isolate in UWW and synthetic BG11 culture medium during a 21-day experiment. (**a**) Growth kinetics plotted using a logarithmic scale. (**b**) Biomass yield expressed as grams of algal dry weight per liter. (**c**) pH trend in culture media. UWW, grey dashed lines or histograms; BG11, black lines or histograms. Data refer to means ± standard deviations (n = 3). In all cases, differences between samples in BG11 and UWW were always significant (*p* < 0.05), except at the inoculation time (time 0).

Parameters linked to the growth of microalgal culture are reported in Figure 3. As concerns the growth kinetics, it is clearly evident that the employment of UWW as the culture medium strongly promoted algal growth (Figure 3a). Different from controls, which showed an initial 4-day lag phase, microalgae in the effluent immediately entered the logarithmic phase of growth and reached a cell concentration of 12 × 10^6^ cells mL^−1^ at the 7th day of experiment (vs 4 × 10^6^ cells mL^−1^ for controls in synthetic BG11 medium). Subsequently, from 7 to 14 days of cultivation, a short and transient stationary phase was observed for both UWW and BG11 algae. In both cases, the temporary stationary phase was followed by a small rise in growth, which resulted in a final cell density of around 8 and 20 × 10^6^ cells mL^−1^, respectively, for samples in synthetic medium and in UWW. On the whole, two growth phases characterized the cultures: a first, evident one from 0 to 4 days for treated algae and from 4 to 7 days for controls (respectively with growth rates, µ, of 0.518 and 0.305 day^−1^; p = 0.0504), and a second, residual one from 14 days to 21 days (µ = 0.09−0.08 day^−1^). Based on these differences, the cell density of algae cultivated in the sewage was always significantly higher than in the control synthetic medium (around 2.5 times at the end of experiment). In parallel, the dry biomass data gave results in agreement with the observed growth kinetics, but a more emphasized difference between control and UWW-treated samples was observed (Figure 3b). Indeed, starting from 4 days of cultivation up to the end of the experiment, the dry biomass yield of treated samples was around 5–6 times higher than that of controls in BG11, the highest difference being at the 4th and 7th experimental day (6 times) (Figure 3b). On the basis of dry biomass results, for the time interval 0-21 days, a mean daily productivity of 23 mg _DW_ L^−1^ d^−1^ for UWW and of only 3.2 mg_DW_ L^−1^ d^−1^ for controls in BG11 was calculated.

As concerns pH values, they obviously increased in UWW cultures from 7.5 at time 0 to 9.7 at 21 days of cultivation; conversely, pH remained at levels lower than 6.6 in BG11 samples soon after 4 days of experiment and reached the lowest value of 5.3 at the end of the experiment (Figure 3c).

#### 2.2.2. Comparative Nutrient Removal in UWW and Modified BG11

**Figure 4:**
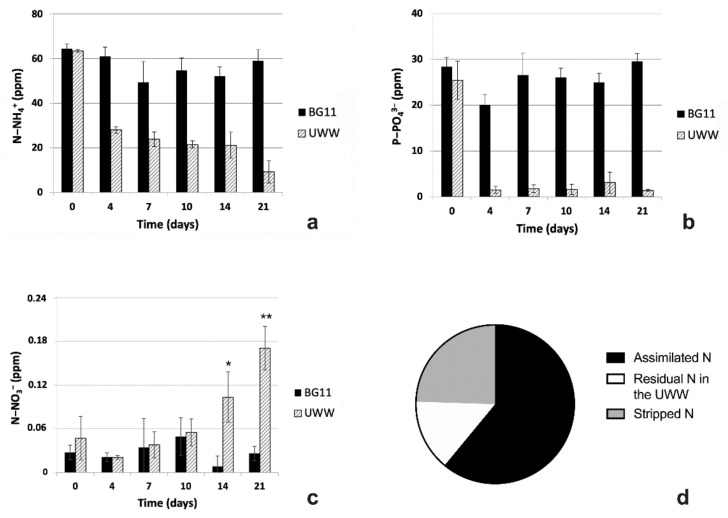
Trend of nutrients (N-NH_4_^+^, P-PO_4_^3-^ and N-NO_3_^-^; ppm) removal and percentage estimate of N mass balance. (**a**) N-NH_4_^+^, (**b**) P-PO_4_^3-^ and (**c**) N-NO_3_^-^ trends in UWW and in synthetic BG11 culture medium during 21-day experiment. (**d**) Estimate of N mass balance in UWW samples at 21 days of experiment. In (**a**–**c**), UWW, grey columns; BG11, black columns. (**d**) In pie graph, % of N assimilated by microalgae, black; % of N residual in the UWW, white; % of stripped N, grey. Data refer to means ± standard deviations (n = 3). In (**a**) and (**b**), differences between samples in UWW and BG11 were always significant (*p* < 0.05), except at time 0. In (**c**), asterisks identify significant differences: *, *p* ≤ 0.05; **, *p* < 0.01.

During the 21-day experiments, BG11 media did not undergo relevant removal of nutrients, with a percentage removal efficiency (RE) of only around 8% for ammonium and no abatement for phosphates. Only a transitory removal was observed during the first periods of cultivation, with a RE of 19% for ammonium from 4 to 7 days of cultivation and of 29% for phosphates from 0 to 4 days (Figure 4a,b). Differently, UWW media showed a pronounced abatement of both ammonium and phosphates (RE = 85.5% and 94.5%, respectively) (Figure 4a,b). Even for these samples, it is noteworthy to highlight that the most evident removal of both nutrients occurred during the first days of the experiment, i.e., in the time interval 0–4 days. In fact, ammonium concentration was reduced from 63.5 ppm at time 0 to 28 ppm at 4 days (RE = 56%) and phosphates from 25.3 to even 1.5 ppm in the same time interval (RE = 94%). Therefore, almost all P-PO_4_^3−^ was removed in only 4 days of cultivation, while a similar N-NH_4_^+^ abatement required a longer period of time (21 days).

Concerning N-NO_3_^−^, in BG11-modified media, it remained at trace concentrations (below 0.05 ppm) (Figure 4c). Instead, it was noteworthy that the content of this nitrogen form tended to increase in UWW samples, starting from the 10th day of the experiment. In particular, although the samples were subjected to strong variability, an upward trend was still detectable in UWW, from 0.054 to 0.10 and finally to 0.17 ppm, respectively, at 10, 14 and 21 days of experiment.

The paramount growth, parallel to the ammonium removal by UWW cells, enabled us to obtain sufficient biomass for protein content analysis and allowed an estimate of N mass balance. The protein content in cells of 44% of dry weight (DW) corresponded to an estimate of around 11.6 mg of N fixed in a 300 mL culture of the alga, which started from 19.1 mg of N-NH_4_^+^ in the same volume of UWW. Considering a final residual amount of 2.8 mg of N-NH_4_^+^ (14.5% of the starting value), the largest amount of N (61%) was assimilated by the algae, while the missing 24.5% was stripped in the atmosphere (Figure 4d).

#### 2.2.3. Comparative Morphology of the Microalgal Isolate in UWW and Modified BG11

**Figure 5:**
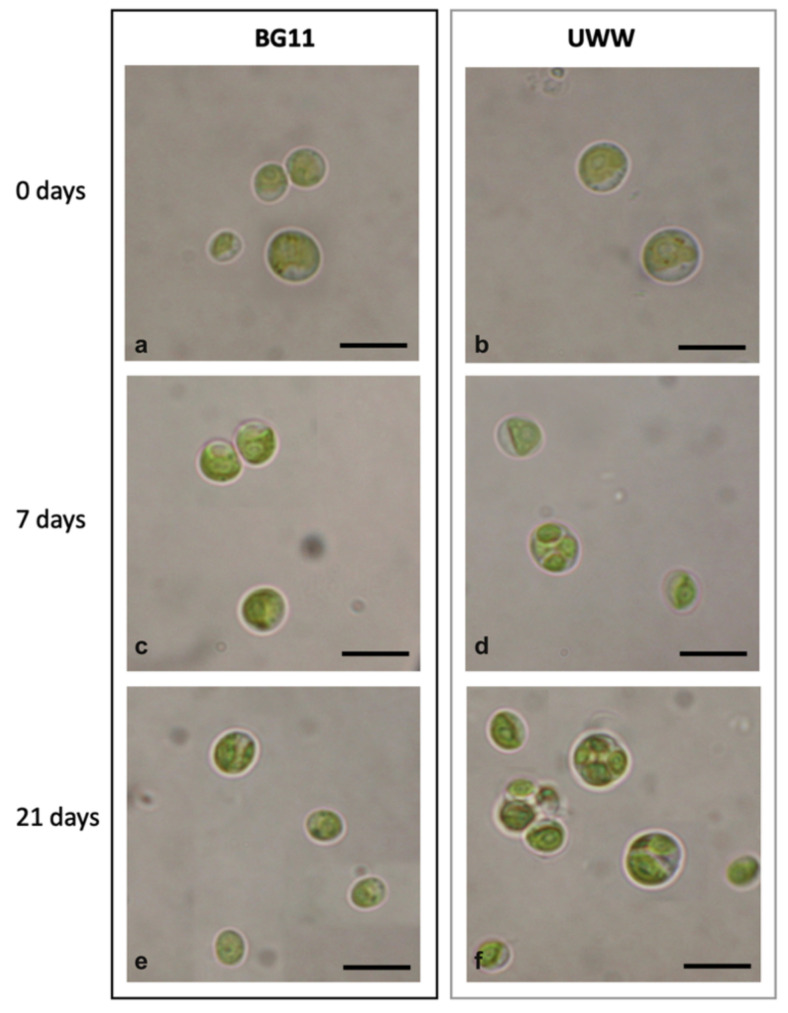
Light microscopy view of microalgae cultivated in modified BG11 and UWW at 0, 7 and 21 days of cultivation. (**a**,**c**,**e**) Cells in synthetic BG11 medium. (**b**,**d**,**f**) Cells in UWW effluent. Bars, 4 µm.

**Figure 6:**
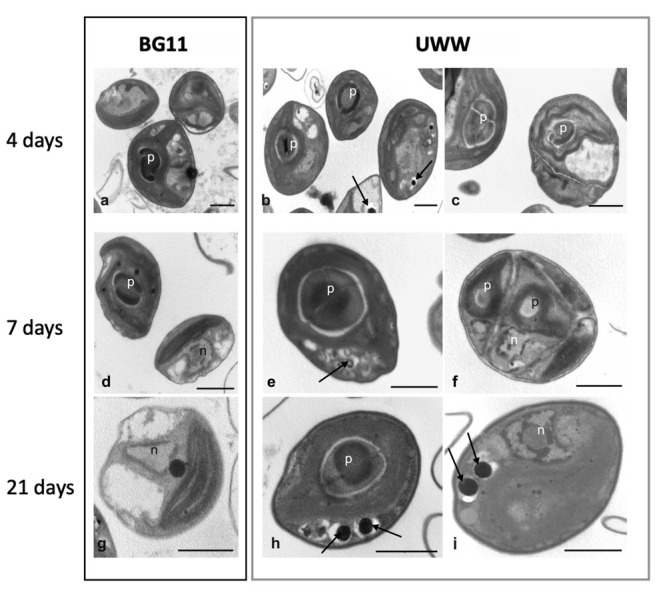
Transmission electron micrographs of microalgae cultivated in modified BG11 and UWW at 4, 7 and 21 days of cultivation. (**a**,**d**,**g**) Cells grown in BG11 synthetic medium and (**b**,**c**,**e**,**f**, **h**,**i**) in UWW effluent for 4 (**a**–**c**), 7 (**d**–**f**) and 21 (**g**–**i**) days. In (**c**) and in (**f**), a dividing cell and a sporocyst are visible, respectively. p, pyrenoid; n, nucleus; arrows, dark polyphosphate precipitates. Bars, 1 µm.

Cultivation of the microalgal isolate in UWW or in modified BG11 did not cause noticeable differences in the cell morphology, since the algae maintained an overall spherical shape, almost similar size (3–5 µm) and a chloroplast with an evident pyrenoid (Figure 5). However, besides these features, cultures in UWW showed frequent sporocysts and dividing cells, already starting from 4–7 days of cultivation and nearly absent in BG11 media (Figure 5, Figure 6). Interestingly, in UWW-treated samples, some bacteria, never observed in BG11-cultivated cultures, could be found (Figure 5 and Figure S1). Ultrastructural observations, instead, highlighted some interesting differences between algae cultivated in synthetic media or in the effluent. As shown in Figure 6a,d, algae in the BG11 control medium were characterized by cells with a regular feature and containing a typical large chloroplast with a well-conformed pyrenoid. With time, these algae showed some evident plastoglobules at 7 days of cultivation, which remained still visible also at 21 days (Figure 6d,g). Finally, at the end of the experiment, BG11-cultivated algae contained large vacuolations and dark cytoplasmic globules, whose nature probably did not refer to polyphosphates, since P was not removed from the medium and, very likely, not even conspicuously absorbed and accumulated by the cells (Figure 6g). Differently, as also emerged by light microscopy, cultures in UWW contained algae with a regular shape and a large chloroplast but showed the occurrence of sporocysts already at the 4–7th day of cultivation (Figure 6). Moreover, the pyrenoid was often very conspicuous, with a large surrounding starch shell (Figure 6b,c,e,f,h). Interestingly, at the cytoplasm level, dark depositions, clearly referrable to polyphosphates, began to accumulate starting from 4 days of cultivation (Figure 6b). These deposits became further evident at the end of the experiment, when also some plastoglobules appeared at the chloroplast level (Figure 6h,i). Lipid globules were never observed in BG11 controls or in UWW-treated algae (Figure 6).

#### 2.2.4. Comparative Photosynthetic Properties of the Microalgal Isolate in UWW and Modified BG11

**Figure 7:**
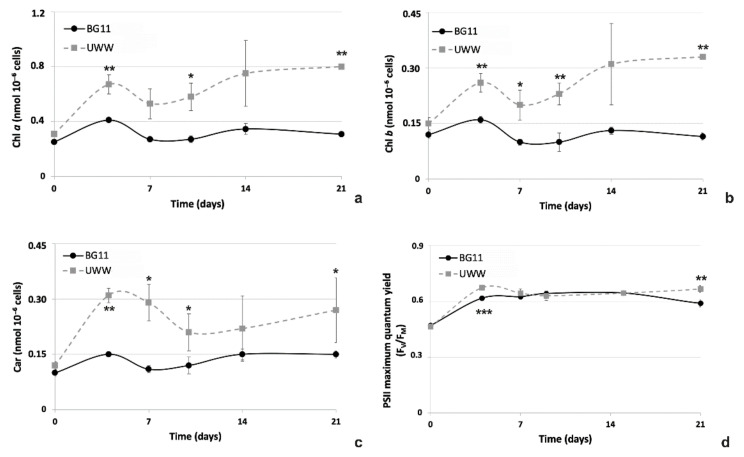
Photosynthetic pigment content (nmol_pigment_ 10^−6^ cells) and PSII maximum quantum yield (F_V_/F_M_ ratio) of microalgae cultivated in UWW and modified BG11 for 21 days. (**a**) Chlorophyll *a*, (**b**) Chlorophyll *b*, (**c**) Carotenoids and (**d**) PSII maximum quantum yield. UWW, grey dash line; BG11, black line. Data refer to means ± standard deviations (n = 3). Asterisks identify significant differences between samples: *, *p* ≤ 0.05; **, *p* < 0.01; ***, *p* < 0.001.

Photosynthetic pigment content was evaluated to obtain two sets of information: the first about the physiological status of the cultures and the other about the biochemical composition of the cells, in view of a potential valorization of the algal biomass after phytoremediation. As immediately evident by observing the graphs of the photosynthetic pigment content (Figure 7a–c), all pigments, chlorophyll (Chl) *a*, Chl *b* and carotenoids (Cars) were more accumulated inside algae cultivated in UWW than in BG11. In particular, trends in concentrations of both Chls were substantially identical between BG11 controls and UWW-treated samples, but soon after 4 days of cultivation, the cells in the effluent reached higher pigment concentrations than in synthetic medium (from +75% at day 4 up to 2.2 times more at the end of the experiment). Differently, for Car content, differences were most conspicuous at 4–7 days of cultivation, when algae in UWW showed a Cars concentration that was doubled in comparison to BG11 (Figure 7c). During the following time interval (10–21 days), differences, though evident (+86%), were minor.

The photochemical activity of PSII (F_V_/F_M_ ratio) of algae remained quite similar in all samples or even slightly higher in UWW samples than in BG11 (Figure 7d).

## 3. Discussion

The presence of nutrients, such as N and P, in UWWs is a well-known problem associated with eutrophication of natural surface waters [7,40,49]. The occurrence of these nutrients is commonly related to human metabolic activities and to the employment of fertilizers or household cleaning products [50,51,52]. In traditional WW-TPs, N is commonly reduced by biological “nitro-denitro” activities of microbial consortia [53,54], while the addition of chemicals, such as sodium aluminate, ferric chloride or calcium hydroxide, is usually also necessary for effective P abatement, but with increasing costs and sludge production as side effects [55,56]. Thus, alternative technologies, including the algae-based ones, for nutrient removal, especially of P, from effluents derived from WAS treatments, are increasingly studied to make the whole depuration process in WW-TPs more efficient and eco-friendly [55,57]. In the WW-TP of Ferrara, sodium aluminate is added directly into the “nitro-denitro” ponds to improve P removal from waters, but with increased production of WAS, still enriched in P, thus requiring water-consuming depuration treatments.

In the present paper, microalgae were isolated or collected from streams of the thickening step of WAS. It is now widely recognized that, compared to microalgae from collections, algae directly isolated from wastewater are potentially better suited to grow in it, exploiting the mineral component for growth and being already adapted to the toxic elements that they may contain (both chemical contaminants and biological competitors) [7,38,40]. However, not all isolates are characterized by growth capacity and nutrient assimilation properties suitable for their profitable use as organisms for phytoremediation; thus, the study of algal isolates is a basic step towards their subsequent employment in WW-TPs, especially in view of the setup of specific customized algae-based phytoremediation plants. From this perspective, even if the addition of CO_2_ is common in algal cultivations, we preferred to not supply CO_2_ to reflect closely the growth condition that could be proposed in a pilot plant to be installed at the WW-TP of Ferrara under the current situation. Taking these considerations into account, several strains have been isolated or separated and studied in this research. Ultrastructural observations suggested that the algal consortium sample harvested from the UWW, containing large-sized algae with a cytoplasm strongly predisposed to the accumulation of polyphosphate granules, could be of interest for phytoremediation. Unfortunately, it was not possible to isolate the single microalga found in the consortium, nor was the consortium able to sufficiently grow in the three growth-tested media (modified BG11, Bristol GR+ and MA) and therefore it was not used for subsequent studies in the present research. Additionally, the consortium was not the most representative algal population in the UWW. Differently, the most abundant alga grown in the UWW was successfully isolated, leading to a monoculture of *Chlorella*-like chlorophyte, which was selected for further studies, even though it did not show apparent morphological characteristics that could suggest an alga capable of accumulating P when cultivated in synthetic medium. In a very attractive way, this isolate gave instead excellent results, both in terms of growth and nutrient removal from the UWW but also of the accumulation of biotechnologically valuable molecules.

Comparing results from cultivations in BG11-modified synthetic medium and in UWW, there is no doubt that the alga clearly preferred the effluent and thus the matrix from which it was isolated. This is evident not only by considering growth and morpho-physiological aspects but also nutrient removal capability.

The growth performance of the isolate in the UWW effluent was surprisingly higher than that of controls and showed a growth rate in line with 6 of the 18 strains tested by Bohutskyi and colleagues [40] in secondary effluents. In detail, in [40], they found that six of the strains that they studied had growth rates similar to that recorded for our isolate (0.518 day^−1^), nine had lower (below 0.3 day^−1^) and only three higher values (0.7 day^−1^). In our work, the effective growth of algae cultures in UWW testifies that N and P content only partially contributed to sustaining the growth of the microalgae. It is plausible that micronutrient composition, together with organic matter responsible for COD and BOD-5 (respectively, 222 and 95 mg L^−1^ O_2_ in the effluent), could have contributed to enhancing the growth of the microalgae in the UWW. Organic matter associated with COD (or BOD-5) is in fact usable for microalgal growth, so much so that microalgae-based systems are proposed for the concomitant removal of N and P and the reduction of COD (or BOD-5) in the depuration treatment of wastewaters, including waste effluents recirculating inside urban WW-TPs [33,58,59,60]. In this regard, a mixotrophic metabolism of the algal isolate could have been activated, as also described for other microalgae, including *C. vulgaris* or *Galdieria sulphuraria*, in wastewater treatment tests [60,61]. As is well known, under mixotrophic conditions, many microalgae can make simultaneous use of both organic and inorganic carbon as carbon sources and of light or organic carbon as energy sources, thus leading to higher growth than autotrophic controls [61]. Moreover, one should take into account that, in the present work, unsterilized UWW was employed, suggesting a co-operative action by the microalgae and the bacteria naturally resident in the effluent, which could have oxidized the organic matter into inorganic compounds (CO_2_ and carbonates), then used by the microalgae for photosynthesis, as also described in [62]. The complex metabolism of microalgae and possible interacting bacteria needs further investigation, but it may explain the effective growth observed in UWW, even in the absence of added CO_2_. Overall, the C:N:P stoichiometry ratio in UWW was clearly more suitable for algal growth than that in the BG11-modified control medium, where C appears to be the limiting factor. In addition, concentrations of other macronutrients and of micronutrients, such as Cu, Mg, K, Mn, Fe, Ca, are never limiting for microalgal growth in effluents [8,10,63]. For example, in the UWW, Cu was 0.052 mg L^−1^, thus similarly abundant as in BG11 (0.02 mg L^−1^) (Table 1) and sufficient to participate in regulatory proteins and metalloenzymes of several metabolic pathways [64,65] and to contribute to the production of organic molecules, including Chls [66]. In general, there is no indication that in our experiment any micronutrient was limiting. The strong accumulation of Chls is particularly indicative of no limitation in Mg. Accordingly, even if it was not measured in the present research, Mg was reported to be around 20 mg L^−1^ in primary and secondary effluents from municipal WW-TPs [8]. On the basis of these considerations, it is inferred that the composition of UWW used for the present study is suitable to guarantee not only N, P and carbon for biomass growth, but also all the micronutrients needed for the remarkable Chls content recorded for UWW-treated microalgae.

Even if P is not a constituent of Chls, its presence in culture media positively impacts the biosynthesis of these pigments as well as the entire energetic metabolism inside the cells [67]. As reported for the microalga *Isochrysis galbana*, the reduction in Chls content in P-limited microalgal cultures cannot be attributed to a nutrient recycling metabolism but to an incapability of synthesizing the ATP required for cell metabolism, including the biosynthesis of Chls [67,68]. In our work, the presence inside UWW-treated cells of polyphosphate granules, as observed with TEM analyses, suggests that, in the effluent, there were the suitable conditions for P assimilation and accumulation by the alga for subsequent metabolic use. In order to balance the observed Chl accumulation and thus maintain the good structure of the photosynthetic machinery, it is reasonable that stores of P could have been also employed to give the algae energy not only for the biosynthesis of Chls but also of Cars [68]. Indeed, in UWW algal samples, the concentration of both photosynthetic pigments was around two times higher than in synthetic BG11 control cells and was accompanied by substantially identical or slightly higher values of F_V_/F_M_ ratio. Moreover, higher values of the aforementioned photosynthetic parameters obtained for UWW algae could represent a further clue supporting the mixotrophic metabolism of the algal isolate in the effluent, as similar results were obtained for a well-known mixotrophic green microalga, *N. oleoabundans*, cultivated with a pure organic carbon substrate [21,69]. Under mixotrophic conditions, the photosynthetic activity is reported to increase, because the organic carbon is used for respiration, releasing CO_2_, which is then used for photosynthesis [70]. The increase in pH observed only in UWW-treated samples, but not in BG11 controls, supports this observation. Differently, in controls, lower growth, accompanied by negligible nutrient reduction in the medium, but by pH decrease, was a sign that in the synthetic medium, algae did not perform sufficient photosynthesis, so respiration produced more CO_2_ than used for photosynthesis, and this resulted in the gradual pH decrease.

Besides pigments, cells grown in the UWW also contained high amounts of other interesting compounds, such as starch and proteins, but not lipids. Ultrastructural observations showed that the algae in the effluent accumulated somehow more starch than control cells in the BG11 synthetic medium; accordingly, pyrenoids in UWW-treated algae were slightly larger than in controls. Differently, neither in the effluent nor in BG11 did the algae accumulate lipids, as revealed by ultrastructural analyses. This is not so surprising, since it is well recognized that lipids are accumulated inside cells in response to nutrient starvation [11,20], a condition that was never detected during this experiment, while starch is the first carbon storage accumulated inside cells, especially under mixotrophic conditions [21]. Because of the importance of proteins and starch for nutrition and of Chls and Cars as antioxidant agents, the biomass obtained using UWW as culture medium for the isolated microalga can be proposed for food/feed supplement production [67,71].

As regards the evaluation of nutrient removal by the microalgal isolate from the effluent or from synthetic media, the very high growth of microalgae in UWW cultures was linked to the conspicuous ammonium and phosphate abatement from the medium (RE, 85.5% and 95.5% for N-NH_4_^+^ and P-PO_4_^3−^, respectively, in 21 days). In parallel, the comparatively modest growth of algae in modified BG11 control medium did not cause any evident nutrient removal.

For P abatement in the UWW effluent, a luxury P uptake mechanism can be proposed, as large polyphosphate depots were formed during cultivation. Luxury P uptake is intended as the strategy that makes algae able to assimilate more P than that needed for immediate growth through the formation of inorganic polyphosphate granules [45,72]. These cellular deposits of P are then metabolized by the alga once P becomes necessary [73,74]. The formation of this cellular P storage represents a promising resource for the utilization of P-enriched algal biomass as P biofertilizer, also in view of the recovery and recycling of this precious element from wastewaters [45,72]. Indeed, it is known that the release of P by biomineralization of polyphosphate granules is sufficiently slow to guarantee efficient absorption by crop plants, thus also reducing the problem of P waste production through washout typical of conventional mineral P fertilizers [75,76]. Moreover, P fertilizer is not renewable, and a global limitation of this element is expected to occur in the next few years owing to its mineral origin [13,49,77]; thus, valorization of waste P through microalgae represents a promising result. Consistent removal of phosphate and ammonium from the effluent reflected assimilation by the algal isolate, also considering that the highest RE of both N-NH_4_^+^ and P-PO_4_^3−^ was obtained already at the 4th day of cultivation, so during the exponential phase of growth of the algae, and when pH of the medium was still below 9, thus excluding a consistent loss of N and P due to stripping or precipitation, respectively, in the 0–4 days interval [78]. Furthermore, the high biomass, around five times more than in modified BG11 synthetic media at the end of the experiment, with a 44%DW of total protein content of UWW-cultivated microalgae, testified to the high assimilation of N inside the algae (61% of the N removed from the UWW during 0-21 days of cultivation was assimilated into the algal biomass). Together with this evidence, it should be mentioned that the pH increase in the medium could have also contributed to the strong abatement of both N and P in the effluent. Solely microalgae cultivated in the effluent grew so efficiently that the pH of the medium significantly increased, especially after the 4th day of cultivation. It is known that, in growing algal cultures, the pH of the cultivation medium increases because of the photosynthetic activity of the algae, which produces OH^−^ while consuming HCO_3_^−^ ions [36,79]. Differently, when algae produce more CO_2_ with respiration than that used for photosynthesis, the pH decreases, as noted in BG11-cultivated cultures. In detail, it is known that pH levels over 9 (as for UWW samples after 4 days of cultivation) result in ammonium stripping or phosphate precipitation [78]; this limits the possibility of chemical-physical removal only during the 4–21 days growth interval, which is, in any case, characterized by a lower RE than the 0–4 days interval.

Parallel to the assimilation of N by microalgae, another explanation for the remarkable N-NH_4_^+^ reduction observed in UWW, but not in BG11 samples, could be very likely attributed to nitrifying bacteria present only in the UWW. Despite its quantitatively negligible presence in the effluent, N-NO_3_^-^ content was measured to possibly highlight interactions between microalgae and bacteria naturally resident in the UWW, so interactions were certainly absent in the controls cultivated in bacteria-free synthetic BG11 medium. Results showed that in UWW samples, the unexpectedly increased concentration of N-NO_3_^−^ could be due to some nitrifying bacteria still present in the effluent and able to convert ammonium into nitrate [33,36]. UWW was not sterilized before use, thus enabling the growth of residual bacteria, as also proposed above to support organic matter oxidation. In WW-TPs, bacteria like *Nitrosomonas* sp. or *Nitrobacter* sp. are often employed in the “nitro-denitro” ponds and their residual presence in the effluent could have contributed to increasing the nitrate content in the sample [43,80]. Concomitantly, microalgae, with their photosynthetic activity, could have enriched water with oxygen, which was then used by bacteria for the nitrification process [36,37].

Overall, our results can support the hypothesis that the conversion of ammonium into nitrate, together with the availability of carbon derived from bacteria activity as reported above, could have contributed to promoting algal growth in the UWW, but not in the synthetic medium, without bacteria. In a preliminary test, in fact, the employment of the UWW alone or inoculated with algae resulted in a different increase in N-NO_3_^−^ (not shown). In UWW alone, an increase in nitrates higher than in UWW with algae occurred, thus making it possible to propose that nitrates produced by nitrifying bacteria activity could have been used by algae, together with ammonium, to support growth. This further opportunity to make use of N by algae, however, did not benefit algae in the synthetic medium, where N-NH_4_^+^ could not be converted into N-NO_3_^−^, thus further explaining the strong difference in growth between algae in modified BG11 and in the UWW.

## 4. Materials and Methods

### 4.1. Wastewater

**Figure 8:**
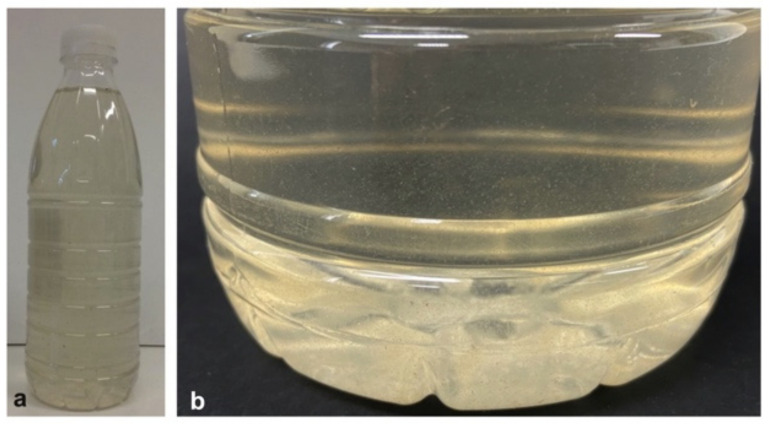
Overall appearance of the UWW used for research. (**a**) Bottle sample with the UWW. (**b**) Detail of the bottom of the bottle with floating clear solid material, after manual shaking.

The substrate employed for the present research was secondarily treated UWW effluent from the HERA SpA (Holding Energia Risorse Ambiente) WW-TP located in Ferrara, Italy (145,000 population equivalent—PE—on BOD basis; 44°51′49″ N, 11°37′47″ E). In January 2019, wastewater was collected from the “sludge line” of the domestic wastewater treatment process, after the thickening step. Part of the UWW effluent was immediately used for the isolation of autochthonous microalgae and the remaining part was stored at −20°C for subsequent experiments on N and P abatement by the isolated microalgae. Low-temperature storage of the UWW was intended to prevent possible alterations due to bacterial proliferation that could occur during the long-lasting isolation step (see Section 4.2). The fresh UWW was quite transparent, with an OD_750_ of 0.050, and contained a solid clear residue that, after freeze-storing, tended to precipitate on the bottom of the bottle (Figure 8). Before algal cultivation for growth and nutrient removal tests, most of the solid clear material, which was mainly composed of bacteria, was manually removed.

The composition of the UWW, determined following standard certified methods used for water quality analyses at the HERA internal laboratories of analysis, is reported in Table 1. N and P contents were not significantly altered by low-temperature storage.

### 4.2. Microalgae Isolation

**Figure 9:**
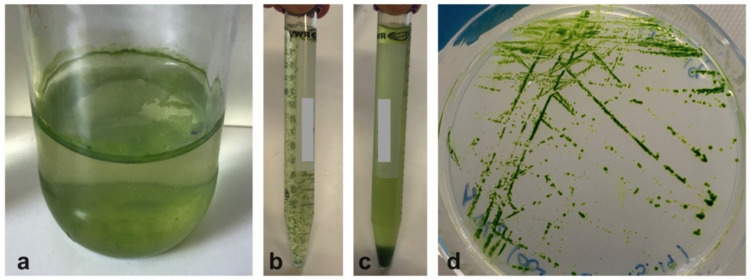
Microalgal material grown in the UWW during the isolation procedure. (**a**) Jar showing two layers containing algal material, an upper one and a lower one. (**b**) Microalgae separated from the upper layer. (**c**) Microalgae separated from the lower layer. (**d**) Example of a Petri dish with isolated colonies of *Chlorella*-like microalgae. No bacteria contamination is visible.

Aliquots of fresh UWW effluent were placed into closed glass jars (150–200 mL volume) and exposed to indirect natural sunlight (PAR, 150–200 µmol_photons_ m^−2^ s^−1^), at room temperature (RT, ranging between 20 and 23°C), for at least 4–6 weeks. During the incubation, microalgal material stratified in two different portions of the jars, on the top and on the bottom (Figure 9a). The algae were quantitatively more abundant in the lower than in the upper level. At the end of the incubation period, all material from both layers was removed and placed into different sterile tubes (Figure 9b,c). Aliquots of 500 µL of algal material grown from both layers were inoculated on sterile Petri dishes containing agarized and sterilized BG11 synthetic medium [81], modified in N and P content according to ammonium and total P concentrations present in the effluent (Table 1). Inoculated Petri dishes were then incubated for a further 4 weeks under the same conditions reported above. Grown colonies were streaked using a sterile technique onto additional sets of Petri dishes containing BG11-modified medium and kept for isolation under the same light and temperature conditions, as for previous phases. This streaking method was repeated until isolation of axenic unialgal cultures was achieved [82].

Cultivation in Petri dishes led to the isolation of colonies only from material harvested from the bottom of the jars. The most dominant algal colonies were then transferred to sterile modified BG11 liquid medium for maintenance and were identified as a *Chlorella*-like Chlorophyta based on morphological observations at both light and transmission electron microscopy, according to standard procedures [26,37,46,47,48].

Both algae harvested from the upper layer and those isolated from the lower layer through serial plating were preliminarily tested for growth using, besides modified BG11, other cultivation media commonly employed for the cultivation of microalgae, Bristol GR+ and MA medium [83,84]. Only the *Chlorella*-like isolate showed promising growth, especially on modified BG11 medium (not shown).

### 4.3. Experimental Design

After isolation of microalgal strains, the most abundant and growing algal form (i.e., the *Chlorella*-like alga) was chosen for further experiments on growth ability and nutrient removal. Experiments were conducted in batch using 500 mL sterilized borosilicate flasks (300 mL culture volume). Algae were inoculated in UWW effluent (treated) or in sterile modified synthetic BG11 medium (control) to obtain a final cell density of around 2 × 10^6^ cells mL^−1^; cultures were maintained in the environmental conditions reported in the previous paragraph and were manually shaken every day. No additional CO_2_ was used [14,26,40,85,86]. Control and treated samples were set up in 3 replicates each. Various parameters (for algae: cell density, specific growth rate, biomass yield and productivity, PSII maximum quantum yield, photosynthetic pigment and total protein content, morphology; for culture media, i.e., modified BG11 and UWW: pH, N and P content) were determined at 0 (inoculum), 4, 7, 10, 14 and 21 days of experiments.

### 4.4. Light and Transmission Electron Microscopy (TEM)

Isolates and harvested microalgae were observed with a Zeiss, model Axiophot, photo-microscope under conventional light. Pictures were taken with a Canon PowerShot S40 digital camera (4 megapixels), mounted on the ocular through a Leica D150 system. Ultrastructure of cells was also investigated using a Zeiss EM910 (Electron Microscopy Center, University of Ferrara, Ferrara, Italy). For TEM observations, cells were harvested through centrifugation (500 *g*, 10 min) and prepared as follows: for fixation and post-fixation steps, glutaraldehyde (3% v/v in phosphate buffer 0.1 M, pH 7.2; 3h, 4°C) and OsO_4_ (2% v/v in the same buffer; 1h, RT) were used, respectively. Then, cells were dehydrated in acetone series and embedded in Araldite resin. Ultrathin sections were finally stained with lead citrate and uranyl acetate [87].

### 4.5. Growth and pH Evaluations

At each experimental time, cell densities of cultures were evaluated with a Thoma’s counting chamber (HBG, Giessen, Germany). Cell densities were plotted on a logarithmic scale to obtain the growth kinetics, and the growth rates were calculated according to Giovanardi and co-workers [88] using the following Equation (1):

µ (day^-1^) = (log_2_*N*_1_ − log_2_*N*_0_) / (t_1_ − t_0_) (1)

where µ is the growth rate, *N*_1_ the cell number at time t_1_, *N*_0_ the cell number at time 0 and t_1_−t_0_ the time interval (days).

For dry biomass, aliquots of samples were filtered through pre-dried and pre-weighed glass-fiber filters (1.2 μm pore size; Whatman GF/C). Filters with cells were rinsed with 20 mL of distilled water, dried for 72 h at 60°C and weighted until they reached a constant weight [89]. The dry biomass data were used to calculate the biomass yield in cultures (g_DW_ L^−1^) and the daily productivity (g_DW_ L^−1^ d^−1^).

At each experimental time, modified BG11 medium and UWW were harvested by centrifugation (2000 *g*, 10 min) for pH measurements. A Jenway mod. 3510 (Stafforshire, Stone, UK) bench pH-meter was employed. pH is, in fact, a useful parameter to monitor algal growth in culture media and is an important regulator for nutrient bio-availability [78,80].

### 4.6. N and P Analysis

For phytoremediation tests, aliquots of culture media, i.e., synthetic modified BG11 and UWW, were harvested by centrifugation (2000 g, 10 min) for N and P quantification. In detail, nitrate (N-NO_3_^−^), ammonium (N-NH_4_^+^) and phosphate (P-PO_4_^3−^) were quantified colorimetrically using a flow-injection autoanalyzer (Flowsys, Systea SpA, company, Roma, Italy) [90]. The removal efficiency in percentage (RE, %) was calculated according to the following Equation (2):

RE, % = [(C_0_ − C_1_)/C_0_)] 100% (2)

where C_0_ and C_1_ are the nutrient concentration (ppm) at time t_0_ or t_1_, respectively [40].

### 4.7. Photosynthetic Pigment Extraction and Quantification

Cell samples were harvested by centrifugation (8000 g, 15 min), and the extraction of photosynthetic pigments was performed with absolute methanol according to [21]. The extracts were manipulated under dim green light to avoid pigment photo-degradation and were measured with a Pharmacia Ultrospec 2000 UV-Vis spectrophotometer (1-nm bandwidth; Amersham Biosciences, Piscataway, NJ, USA) at 665 nm (Chl *a*), 653 nm (Chl *b*), 470 nm (Cars) and 750 nm (background disturbance). Pigment concentrations were quantified as reported in Wellburn [91] and expressed as μg_pigment_ 10^−6^ cells.

### 4.8. PSII Maximum Quantum Yield Analysis

Concomitant to photosynthetic pigment analyses, the maximum quantum yield of PSII (F_V_/F_M_ ratio) of microalgae was determined using a pulse amplitude modulated fluorometer (Junior PAM, company, Heinz Waltz GmbH, Effeltrich, Germany). Samples were prepared as reported for microalgae in previous studies [21,92]. After 15 min of dark incubation, the basal fluorescence (F_0_) was determined; the maximum fluorescence (F_M_) was measured by flashing the samples with a saturating light pulse (0.6 s). The maximum PSII quantum yield was calculated as F_V_/F_M_ ratio, where F_V_ = F_M_ − F_0_ [93].

### 4.9. Total protein Extraction and Quantification

For total protein quantification, at the end of the phytoremediation tests, aliquots of cultures from the UWW-treated algae were centrifuged for 10 min at 500 *g* and treated according to Ivleva and Golden [94], with some modifications as described in Baldisserotto et al. [21]. No algal biomass from the control samples was harvested for extraction owing to insufficient growth of the culture. After extraction, samples were rapidly frozen in liquid N_2_ and kept at −20°C until quantification. Proteins were quantified following Lowry’s method [95].

An N mass balance estimate was calculated using total protein content as the parameter for algal assimilation of N, assuming a standard 16% N content in proteins [96].

### 4.10. Statistical Analyses of Data

Statistical analyses and graphical representations were routinely performed with Microsoft Office Excel 365. Data were compared using Student’s t-test with significance threshold set at *p <* 0.05. Data are expressed as means ± standard deviations (s.d.) for *n* number of samples (n ≥ 3, depending on analysis). Asterisks in graphs are used to identify the levels of significance: *, *p ≤* 0.05; **, *p ≤* 0.01; and ***, *p ≤* 0.001.

## 5. Conclusions

Results on the abatement of N-NH_4_^+^ (the main N form) and of P-PO_4_^3−^ in the UWW effluent used for cultivation of our microalgal isolate are noticeable (mean content of ammonium and phosphates at 21 days, respectively, 9.2 and 1.4 ppm). National and EU regulations for safe water discharge in natural surface waters with low autodepurative capacity (as is the case of the river which receives waters from the WW-TP of Ferrara) after urban wastewater treatment defines total N and total P concentrations, respectively, below 10 and 1 mg L^−1^, i.e., ppm [3]. Therefore, it is absolutely clear that the treated effluent, almost nutrient-free, can be recirculated in the WW-TP, helping to improve its efficiency (for example, by reducing freshwater use for depuration phases of WAS), while for a removal completely meeting law limits for safe disposal in the environment, longer cultivation could be proposed (1 additional week would likely be sufficient).

The abatement of N-NH_4_^+^ and P-PO_4_^3−^ from UWW could be due to a combination of different modes linked to the treatment of the effluent with the microalgal isolate: 1. microalgal assimilation, 2. interaction with other microorganisms and 3. chemical-physical effects. Integration into the microalgal biomass was the most relevant.

Moreover, the algal isolate, while removing nutrients from the UWW effluent, produced a high biomass, rich in biotechnologically valuable compounds, such as Chls, Cars, starch and proteins, thus suggesting its profitable use as food/feed supplements or, even better, as biofertilizers [97]. Instead, the employment of the microalgal biomass in the bioenergetic sector, which is mainly proposed for lipid-rich microalgae derived from wastewater treatments [1,2,9], should be limited to energy production in anaerobic biodigestors, such as those present in the majority of conventional WW-TPs [5].

On the other hand, cultivation in BG11-modified medium, which did not significantly influence the algal characteristics, could be proposed for maintaining the microalgae as stock cultures to be employed as inocula in case of loss of microalgae in UWW, a possible event especially if applying microalgal technology to WW-TPs. Related to their origin, the composition of domestic wastewaters is subjected to strong daily and seasonal variations, which could impact negatively on algal growth [98,99]. From this perspective, forthcoming research will be aimed at testing the metabolism flexibility of the algal isolate in UWW effluents harvested in different seasonal periods, for employment in a microalgae-based innovative pilot-scale phytoremediation plant at the HERA-Ferrara WW-TP.

## Figures and Tables

**Table 1:** Characterization of the UWW and analytical methods employed for the analyses.

Parameter	Unit	Analytical Method	UWW
Total nitrogen	mg N/L	UNI EN 12260:2004	61.8
Ammonium	mg NH_4_ – N/L	APAT CNR IRSA 4030 A1 Man 29 2003	66.5
Nitrate	mg NO_3_ – N/L	APAT CNR IRSA 4020 Man 29 2003	<0.5
Nitrite	mg NO_2_ – N/L	APAT CNR IRSA 4050 Man 29 2003	<0.04
Total phosphorus	mg P/L	UNI EN ISO 15587-2:2002 + UNI EN ISO 17294-2:2016	24.8
BOD-5	mg O_2_/L	APHA Standard Methods for the Examination of Water and Wastewater ed 23rd 2017 5210	95
COD	mg O_2_/L	ISO 15705 par 10.2:2002	222
Cr	mg/L	UNI EN ISO 15587-2:2002 + UNI EN ISO 17294-2:2016	<0.02
Cr (VI)	mg/L	APAT CNR IRSA 3150 C Man 29 2003	<0.02
Cu	mg/L	UNI EN ISO 1187-2_2002 + UNI EN ISO 17294-2:2016	0.052
Hg	mg/L	UNI EN ISO 1187-2_2002 + UNI EN ISO 17294-2:2016	<0.001
Ni	mg/L	UNI EN ISO 1187-2_2002 + UNI EN ISO 17294-2:2016	<0.01
Pb	mg/L	UNI EN ISO 1187-2_2002 + UNI EN ISO 17294-2:2016	<0.005

## Supplementary Materials

The following is available online at https://www.mdpi.com/2223-7747/9/12/1802/s1.. Figure S1: Light microscopy view of microalgae cultivated in UWW at 4, 7 and 21 days of cultivation. Bacterial contamination is shown. Bars, 5 µm.
