# Supplementary material for: Removal of Nitrogen and Phosphorus from Thickening Effluent of an Urban Wastewater Treatment Plant by an Isolated Green Microalga
Source: Plants (Basel). 2020 Dec 18;9(12):1802. doi: 10.3390/plants9121802 (PMC7766996; doi:10.3390/plants9121802)
Supplement: Supplementary file 1 [file plants-09-01802-s001.pdf]

**Supplementary materials:**

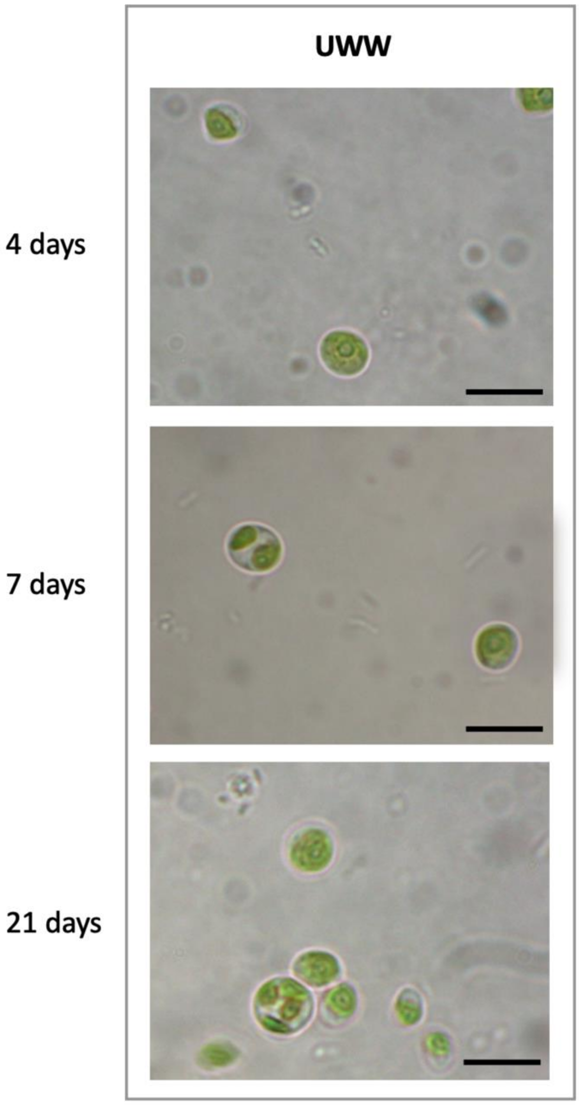


**Figure S1**: Light microscopy view of microalgae cultivated in UWW at 4, 7 and 21 days of cultivation. Bacterial contamination is showed. Bars, 5 μm.
